# Augmented cAMP Signaling by Co-Administration of Resveratrol and Curcumin: A Cellular Biosensor Kinetic Assessment

**Published:** 2019-07

**Authors:** Farnaz SAFAVIFAR, Farshid SAADAT, Seyedeh Zohreh JALALI, Mohammad Reza KHORAMIZADEH

**Affiliations:** 1. Endocrinology and Metabolism Research Center, Endocrinology and Metabolism Clinical Sciences Institute, Tehran University of Medical Sciences, Tehran, Iran; 2. Faculty of Medicine, Guilan University of Medical Sciences, Rasht, Iran; 3. Biotechnology Research Center, Tehran University of Medical Sciences, Tehran, Iran; 4. Biosensor Research Center, Endocrinology and Metabolism Molecular-Cellular Sciences Institute, Tehran University of Medical Sciences, Tehran, Iran

**Keywords:** cAMP, Cellular biosensor, Curcumin, Inflammation, Resveratrol

## Abstract

**Background:**

Curcumin and resveratrol are two polyphenolic compounds extensively investigated for their medicinal effects on inflammatory signaling. However, there is a paucity of information on the Adenosine-3′, 5′-cyclic monophosphate (cAMP) kinetics following administration of curcumin and resveratrol in biological systems. In this study, kinetic modulation of cAMP as a target detection messenger in pro-inflammatory pathways was assessed by co-administration of curcumin and resveratrol using a cellular sensor model.

**Methods:**

To evaluate their putative activity, curcumin and resveratrol compounds were administered alone or in combination on the media culture of cAMP EPAC (exchange protein directly activated by cAMP) bioluminescence resonance energy transfer (BRET) biosensor. The study was performed at the following two centers at Tehran University of Medical Sciences (TUMS): 1- Biotechnology Research Center, and, 2- Endocrinology and Metabolism Research Institute (EMRI) in 2017. Time course kinetic of cAMP response signals were plotted. Forskolin and IBMX were used to stabilize the cAMP signals.

**Results:**

When we treated HEK-293T biosensor cells at 10uM concentration, curcumin and resveratrol upregulated cAMP signaling. Co-administration of resveratrol and curcumin revealed an augmented cAMP level, as compared to treatments with the compounds alone.

**Conclusion:**

Co-administration of curcumin and resveratrol leverage cAMP kinetic response in a time-course manner. The presented methodology can be readily adopted for drug development and novel biopharmaceutical functional analyses.

## Introduction

Inflammation underlies numerous pathological processes that trigger such chronic human diseases as diabetes. Recent studies have shed light on our understanding of inflammatory responses and involved molecules (1–3). Cyclic adenosine monophosphate (cAMP) is a derivative of adenosine triphosphate (ATP) which used for intracellular signal transduction in many different organisms. The importance of the host cAMP axis in regulating inflammatory response encourages searching for modulating components. Curcumin (CUR), the essential constituent of turmeric (*Curcuma langa L*) has long been reported as having multiple health benefits in diabetes, cancers, cardiovascular and autoimmune diseases (4, 5). The therapeutic potentials of this natural polylyphenol agent have been extensively advocated due to its inherent pharmacological safety and efficacy through the suppression of numerous cell-signaling pathways. In an experimental antioxidant model, curcumin has been documented to improve STZ-induced hyperglycemia/glucose tolerance, to ameliorate the damage of pancreatic islets, to prevent type 2 Diabetes (T2D), and to inverse hypoinsulinemia (6, 7). However, despite its health benefits, the molecular targets and mechanisms of action remain abstruse, owing in part to the lack of both controllable bioavailability and kinetic detectability.

Resveratrol (RES; 3, 40, 5-trihydroxystilbene) is a polyphenolic compound found in grapes and other fruits which modulates the transcription factor NF-ϰB (8). Attributable health values for RES have been documented so far. Examples include its chemopreventive mechanism to elevate cAMP release in human breast cancer cells (9) and to protect cardiovascular disease through increased cGMP production in coronary arterial smooth muscle cells (10). Moreover, RES has been shown to possess therapeutic potential in the fight against obesity and T2DM (11). The extent of beneficial effects of RES has also been tested in a number of diabetic models including streptozotocin (STZ), nicotinamide/STZ, and long-term high-fat diets (12–14). RES supplementation improved glycemic control, and insulin sensitivity, and reduced oxidative stress in T2DM patients (15–18).

Bioluminescence resonance energy transfer (BRET) is a naturally occurring phenomenon in which a non-radiative transfer of energy from a donor like an excited luminescent enzyme or substrate to an acceptor occurs (19). In many BRET studies published to date, the donors were variants of the enzyme, *Renilla reniformis* luciferase (Rluc) (20, 21). Degradation of a luminescent chemical substrate, coelenterazine, by Rluc excites the acceptor fluorophore. The light emitting acceptors are variants of green fluorescent proteins.

In the present study, we applied a genetically cell biosensor, which measures intracellular cAMP concentrations in cytosol of living mammalian cells to assess the co-administration of curcumin and resveratrol influence cAMP kinetic response in a time course kinetic manner.

## Materials and Methods

The study was approved by the Research Ethics Committee of the Tehran University of Medical Sciences (code 25336). The study was performed at the following two centers at Tehran University of Medical Sciences (TUMS): 1- Biotechnology Research Center and, 2- Endocrinology and Metabolism Research Institute (EMRI) in 2017.

### Reagents

RES was purchased from Tocris Bioscience (Minneapolis, MN, USA). CUR, 3-isobutyl-1-methylxanthine (IBMX), and all other materials such as forskolin, coelenterazine and 3-isobutyl-1-methylxanthine (IBMX) were fine grade and obtained from Sigma-Aldrich (St. Louis, MO, USA).

### Cell Culture

The wild type HEK-293T cells were purchased from National Cell Bank of Pasture Institute of Iran, Tehran, Iran. The cell line maintained in Dulbecco’s Modified Eagle’s Medium (DMEM) (Invitrogen, Carlsbad, CA) supplemented with 10% fetal bovine serum and antibiotics. Following overnight incubation at 37 °C, cells were collected, plated in culture flask, and allowed to reach confluency at 37 °C in a humidified atmosphere of 5% CO2 the day before the transfection using trypsin-EDTA.

### The BRET-based cyclic AMP biosensor

The EPAC cAMP biosensor relies on BRET which generated by modification of the ICUE2 cAMP FRET biosensor (22). The sensor consists of an N-terminal truncated variant of the EPAC tagged with a donor (*Renilla* Luciferase, Rluc) and a yellow fluorescent protein variant (YFP) attached at the N and C termini, respectively. For BRET studies, the HEK-293T cells were transfected with 3 ug of BRET biosensor EPAC construct cDNA in 1 ml of calcium-phosphate transfection solution (Sigma-Aldrich, USA) and cells which express the EPAC sensor were selected.

### CUR and RES treatments and BRET screening kinetic assays

HEK-293T cells permanently transfected with the EPAC sensor were split into 96-well plates at 15 to 20 × 10^4^ cells per well. After washing with PBS on the following day, PBS (containing calcium and magnesium salt) and coelenterazine solution were added to each well. After 10 min incubation, vehicle, curcumin (10 uM), resveratrol (10 uM) or both were added. The plate was then placed into a Mithras LB940 instrument (Berthold Technologies, Bad Wildbad, Germany) that allowed the sequential integration of the luminescent signals detected in the 465 to 505 nm and 505 to 555 nm windows using filters with the appropriate band pass and by using MicroWin 2000 software (Berthold Technologies). The BRET signal is determined by calculating the ratio of the light emitted at 505 to 555 nm to the light emitted at 465 to 505 nm.

### Statistical analysis

Each experiment was performed three times and normally distributed data are presented as means ± SEM. The differences in cell luminescent were determined using the Student’s t-test by the statistical software SPSS 20 (Chicago, IL, USA). *P* value <0.05 was considered significant.

## Results

### EPAC-transfected cells for measuring cAMP in real time

We evaluated the efficacy of our EPAC-transfected cells biosensor to produce cAMP in real time using forskolin and IBMX as cAMP inducer. Upon binding cAMP, the signal of the biosensor decreases because of a conformational change that presumably increased the distance between the Rluc donor and the yellow fluorescent protein acceptor. EPAC-transfected cells biosensor showed no statistically differences between these repeats in various time courses (F=0.714, 28; *P*>0.845).

### Effect of CUR and RES on BRET cell sensor in real time

**Fig. 1:**
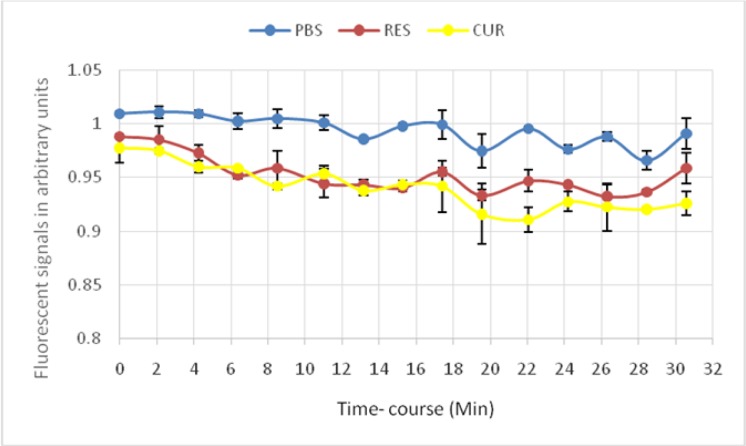
Ratio of the light emitted for CUR and RES in comparison with negative control using EPAC-transfected cells biosensor assay Wells of duplicate rows in the 96-W test plate BRET assay were filled with equal volumes of PBS vehicle alone as blank (upper curve) or RES (mid curve) or CUR (lower curve), added by forskolin and IBMX. The significant shifting down of RES and CUR curves indicates dynamic activation of cAMP signal over time-course experiment. Data are expressed as mean± SD. From top to Bottom: 1- PBS + Forskolin+IBMX; 2-RES (10uM)+Forskolin+IBMX; 3-CUR (10uM) + Forskolin+IBMX

In order to evaluate effect of Curcumin and Resveratrol on cAMP signaling pathway, the mentioned amount of CUR and RES were added. The ratio of the light emitted for curcumin and Resveratrol in comparison with positive and negative components using EPAC-transfected cells biosensor assay is depicted in Fig. 1.

Although significant differences between Cur and Rev compared to control (*P*=0.001, *P*=0.001), there were no differences between CUR and RES (*P*=0.098) based on our statistical analysis. There were no statistically differences between these repeats in various time courses. These compounds produce a sustained reduction in the BRET signal in all time points.

### Effect of CUR+REV on BRET cell sensor

**Fig.2:**
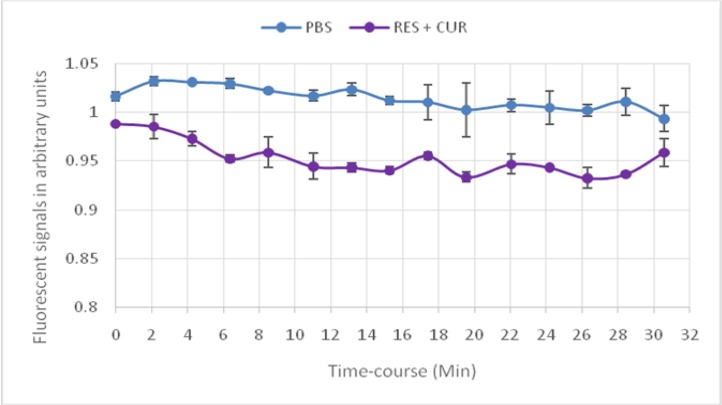
Curcumin+Resveratrol in comparison with negative control using EPAC-transfected cells biosensor assay Combination of RES and CUR (lower curve) significantly raised the cAMP time-course emission signals, as compared to the vehicle PBS blank (upper curve). Data are expressed as mean± SD. From Top to Bottom: 1-PBS+Forskolin+IBMX; 2-PBS+CUR (10uM)+RES (10uM)+ Forskolin+IBMX

**Fig. 3:**
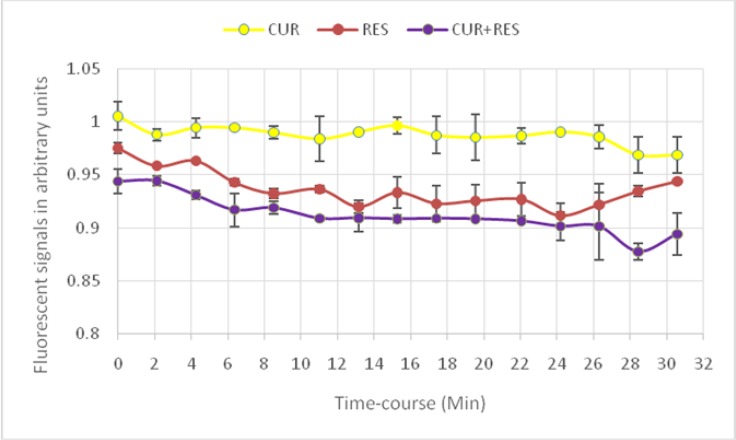
Time course of the BRET sensor response to cAMP accumulation after exposure to 10uM CUR, RES, or CUR+RES Depiction of elevated cAMP activation signal with combined RES and CUR treatment (lower curve), as compared to RES- (mid) or CUR-alone (upper) curves. Data are expressed as mean± SD. From top to Bottom: 1- CUR (10uM)+Forskolin+IBMX; 2-RES (10uM)+Forskolin+IBMX; 3-CUR +RES(10uM) + Forskolin + IBMX

**Fig. 4:**
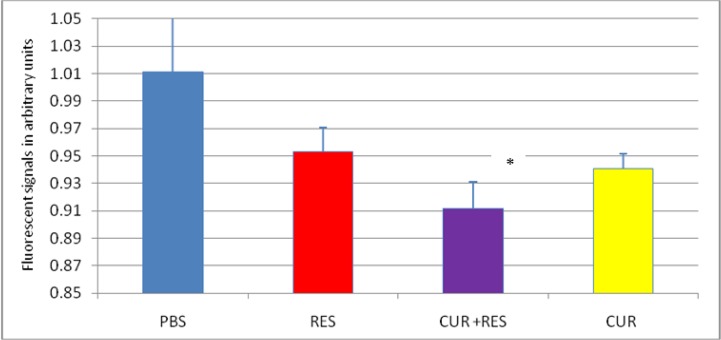
End point of the BRET sensor response to cAMP accumulation after exposure to 10uM CUR, RES, or CUR+RES Comparative end point analysis of RES, CUR, and CUR+RES was depicted to corroborate the time-course kinetic cAMP emission BRET assays. Data are expressed as mean± SD. Significant differences are indicated by *P<0.001 compared with controls

The efficacy of a mixture of CUR and REV on the biosensor for measuring EPAC cAMP signaling in real time is demonstrated in Fig. 2. cAMP production was augmented statistically when a mixture of CUR & RES compare with each of them administration (*P*=0.001) (Figs. 3, 4).

## Discussion

Subclinical inflammation is considered as one of the primary triggers for many pathological circumstances like type 2 diabetes. Although T2D is developed because of β cell dysfunction, the role of cAMP generation in these cells is not completely accepted. Experimental models of diabetes showed a reduction in cAMP induction by glucose which improved by cAMP-elevating agents (23). This plausible mechanism should be influenced by curcumin and resveratrol bioactive compounds with relatively higher safety profiles (7, 12).

The cAMP EPAC biosensor used in our studies was originally developed as a FRET biosensor (24) and later adapted for BRET applications (19). In the lack of cAMP at resting levels, there is considerable basal energy transfer between fluorophores, suggesting the donor and the acceptor remain in adjacent positioning. Whenever cAMP increases, BRET ratios decrease due to a conformational change leading to increased distance between the Rluc and YFP. The main advantage of exchange protein directly activated by cAMP is the possibility to measure the fluctuations of cAMP in Real-time. Moreover, this BRET sensor should evaluate the contribution and the kinetic of different components that modulate cAMP levels.

Based on our findings, curcumin steadily decreases the ratio of the light emitted which means that it increases the cAMP levels at the molecular level. This result in agreement that found increased tissue cAMP levels in liver and less in other adipose tissue except in interscapular subcutaneous fat (25). Because of CUR rapid metabolism in cells, we examined it in short period of time, since the effect of CUR metabolites on cAMP production should be practically faded. Recent studies in mice have revealed that curcumin modulates plasma lipid levels and mitochondrial biogenesis via increasing the level of cAMP (25, 26). Although other signaling pathways including NF-ϰB, STAT3 and Nrf2 involved in ameliorative effect of curcumin in various types of diseases, the cAMP/protein kinaseA pathway also plays a pivotal role in some of them (5).

Other examined polyphenolic compound, resveratrol, is also modulated numerous transcriptional factor like SIRT family which regulate glucose and fat metabolism; Nevertheless, the RES is not biologically effectiveness in human administration (27, 28). Here, we documented that resveratrol increased the level of cAMP. Resveratrol was capable to regulate cyclic AMP response element (CRE) in human embryonic kidney cells (29). Moreover, curcumin had no effect upon CRE-regulated transcription, which persuades its usage with other nutraceuticals such as resveratrol.

Our data showed cAMP production of a mixture of CUR and REV on the EPAC cAMP cell biosensor is augmented statistically significant compared with each of them administration. Taken together, the synergistic effect of these mention combination should be considered as a better protectiveness in cell stress conditions.

## Conclusion

Dual-ingredient formulation of curcumin and resveratrol could dramatically enhance cell induction through cAMP production. This might be considered for new drug development.

## Ethical considerations

Ethical issues (Including plagiarism, informed consent, misconduct, data fabrication and/or falsification, double publication and/or submission, redundancy, etc.) have been completely observed by the authors.
